# Supplementary material for: Karyotype Variability in Wild Narcissus poeticus L. Populations from Different Environmental Conditions in the Dinaric Alps
Source: Plants (Basel). 2024 Jan 11;13(2):208. doi: 10.3390/plants13020208 (PMC10818684; doi:10.3390/plants13020208)
Supplement: Supplementary file 1 [file plants-13-00208-s001.zip › Supplementary captions.pdf]

Supplementary Materials

Figure S1: The activity of the rRNA genes on B chromosomes in *Narcissus poeticus*. (**A**) Silver staining of the nucleoli of an individual with 1B chromosome. Scale bar on photo = 5 μm. (**B**) Confirmation of activity of 35S rRNA genes (red fluorescence) in the representative interphase nucleus after FISH with 1B chromosome (white arrows mark their positions on A and B chromosomes). Scale bar on photo = 5 μm.

Figure S2: Results of chromomycin banding and FISH of *Narcissus poeticus* individuals with chromosomal rearrangements. (**A1**) CMA, (**A2**) FISH and (**A3**) idiogram of an individual from the serpentine population (pop 12) with inversion on pair 4 (arrows, boxed). (**B1**) and (**C1**) CMA, (**B2**) and (**C2**) FISH, and (**B3**) and (**C3**) idiograms of two individuals showing the translocations in the serpentine (B; pop 12) and limestone (C; pop 2; unpaired chromosomes boxed) populations. Scale bar on photos = 5 μm.

Figure S3: 2C cytometric histogram of *N. poeticus* with *Artemisia arborescens* as internal standard.

Table S1: Mean values of morphometric data for the standard haploid karyotype of *Narcissus poeticus* from 13 populations studied. L, long arm; S, short arm; TLC, total chromosome length (TLC = L+S); r, arm ratio (r = L/S); Ci, centromeric index [Ci = 100 x S/(L+S)]; CT, chromosome type (m–metacentric, sm–submetacentric, st–subtelocentric; according to Levan et al. [63]); ΣTLC, total chromosome lengths for haploid karyotype; AsI, asymmetry index (AsI % = (∑L / ΣTL) x 100; according to Arano and Saito [64]); R, ratio longest/shortest chromosome pair; sd, standard deviation; I,II,III, B chromosome types.
