# Supplementary material for: Karyotype Variability in Wild Narcissus poeticus L. Populations from Different Environmental Conditions in the Dinaric Alps
Source: Plants (Basel). 2024 Jan 11;13(2):208. doi: 10.3390/plants13020208 (PMC10818684; doi:10.3390/plants13020208)
Supplement: Supplementary file 1 [file plants-13-00208-s001.zip › Table S1.pdf]

Table S1. Mean values of morphometric data for the standard haploid karyotype of *Narcissus poeticus* from 13 populations studied.

**Chromosome pair**	**1**	**2**	**3**	**4**	**5**	**6**	**7**	**I**	**II**	**III**
L (μm±sd)	6.49±0.27	6.09±0.23	5.95±0.17	5.61±0.39	4.42±0.235	5.89±0.19	3.15±0.19	2.49±0.59	1.89±0.13	2.86±0.21
S (μm±sd)	2.82±0.31	2.78±0.19	2.35±0.24	2.69±0.18	3.21±0.27	1.64±0.18	1.87±0.47	1.05±0.49	1.78±0.19	0.48±0.12
TLC (μm±sd)	9.31±0.36	8.87±0.25	8.30±0.16	8.30±0.31	7.63±0.28	7.53±0.12	5.02±0.21	3.54±0.30	3.67±0.07	3.34±0.11
r	2.30	2.19	2.53	2.09	1.38	3.59	1.68	2.37	1.06	6.00
Ci	30.29	31.34	28.31	32.41	42.07	21.78	37.25			
CT	sm	sm	sm	sm	m	st	m	sm	m	st
ΣTLC	54.96									
AsI	67.05									
R	1.85									

L, long arm; S, short arm; TLC, total chromosome length (TLC = L+S); r, arm ratio (r = L/S); Ci, centromeric index /Ci = 100 x S/(L+S)]; CT, chromosome type [m–metacentric, sm–submetacentric, st–subtelocentric; according to Levan et al. [63]/; ΣTLC, total chromosome lengths for haploid karyotype; AsI, asymmetric index /AsI % = (Σ L / Σ TL) x 100; according to Arano and Saito [64]; R, ratio longest/shortest chromosome pair; sd, standard deviation; I,II,III, B chromosome types.
