# Supplementary figures and images for: Karyotype Variability in Wild Narcissus poeticus L. Populations from Different Environmental Conditions in the Dinaric Alps
Source: Plants (Basel). 2024 Jan 11;13(2):208. doi: 10.3390/plants13020208 (PMC10818684; doi:10.3390/plants13020208)

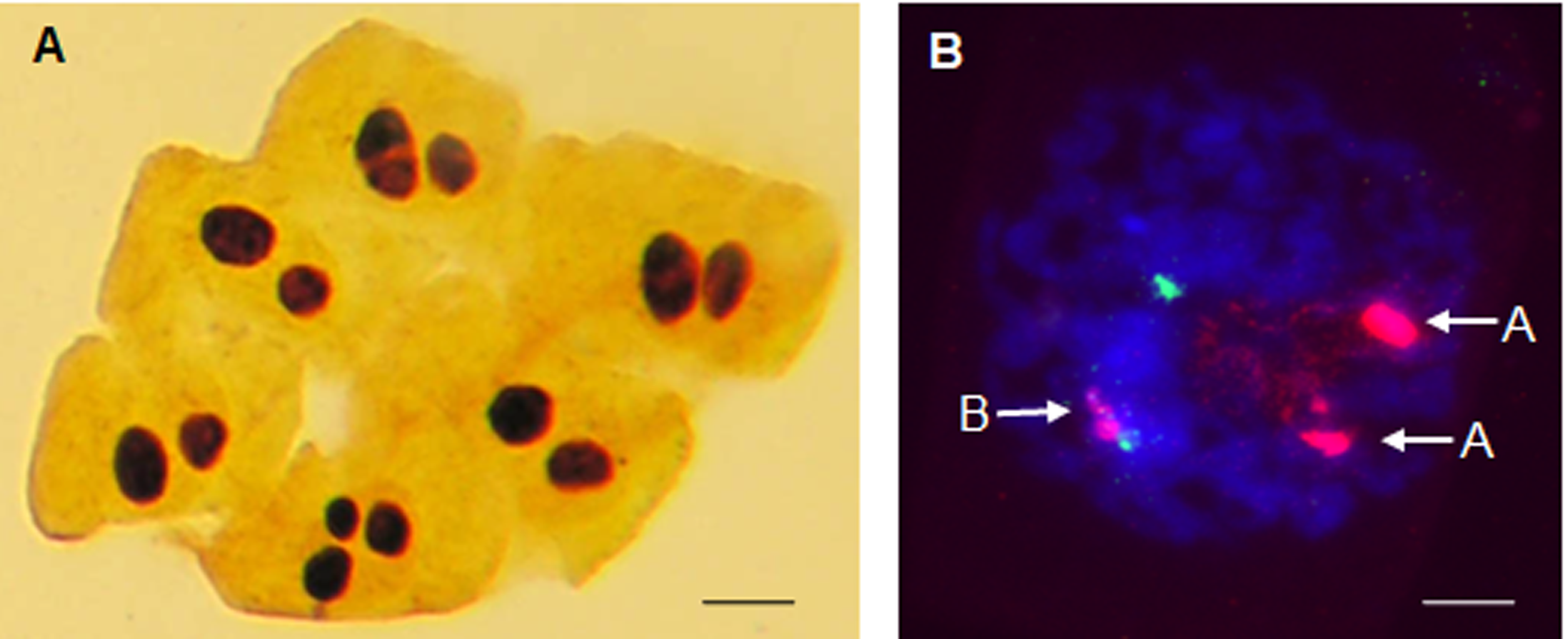

Supplement: Supplementary file 1 [file plants-13-00208-s001.zip › Figure S1.tif]

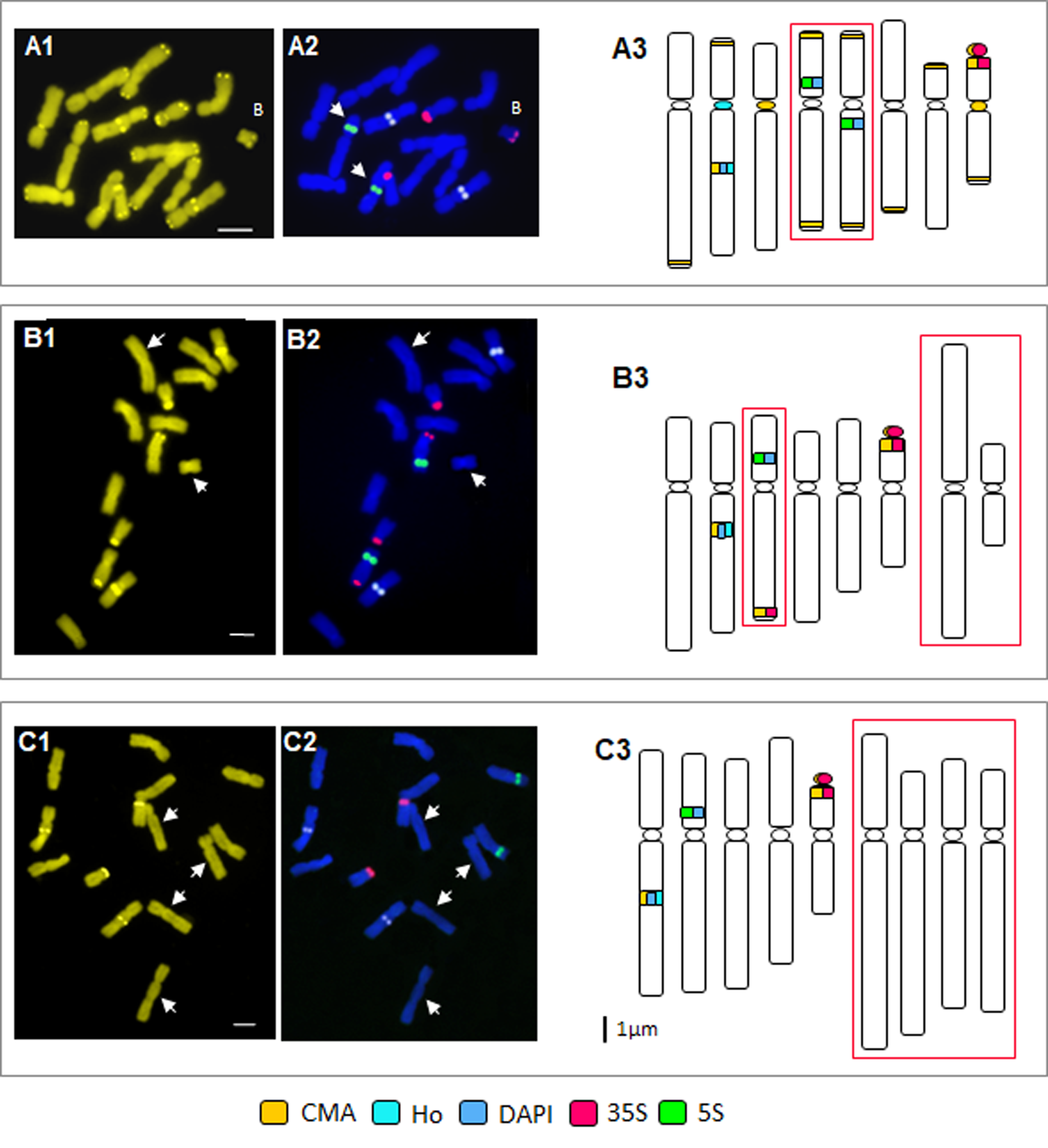

Supplement: Supplementary file 1 [file plants-13-00208-s001.zip › Figure S2.tif]

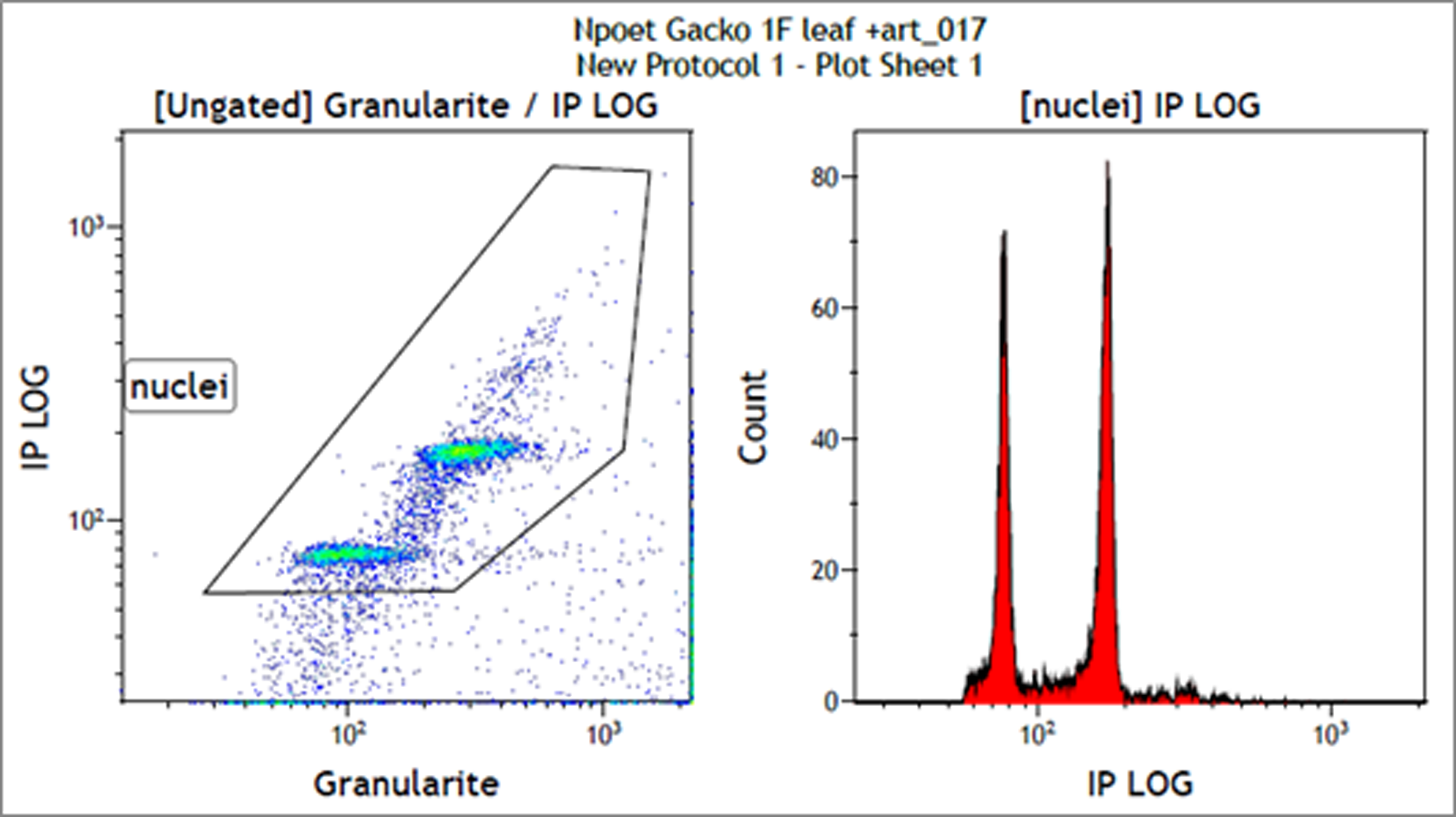

Supplement: Supplementary file 1 [file plants-13-00208-s001.zip › Figure S3.tif]
